# Managing elderly patients with dual metastatic cancers—navigating diagnostic and treatment challenges

**DOI:** 10.1093/oncolo/oyaf026

**Published:** 2025-03-10

**Authors:** Zane Gray, Nicholas Levonyak, Liwei Jia, Richard Ahn, Jyoti Balani, Jue Wang

**Affiliations:** Department of Internal Medicine, University of Texas Southwestern Medical Center, Dallas, TX 75390-8852, United States; Department of Internal Medicine, University of Texas Southwestern Medical Center, Dallas, TX 75390-8852, United States; Hematology-Oncology Division, Department of Internal Medicine, University of Texas Southwestern Medical Center, Dallas, TX 75390-8852, United States; Department of Pathology, University of Texas Southwestern Medical Center, Dallas, TX 75390-8852, United States; Department of Radiology, University of Texas Southwestern Medical Center, Dallas, TX 75390-8852, United States; Department of Pathology, University of Texas Southwestern Medical Center, Dallas, TX 75390-8852, United States; Department of Internal Medicine, University of Texas Southwestern Medical Center, Dallas, TX 75390-8852, United States; Hematology-Oncology Division, Department of Internal Medicine, University of Texas Southwestern Medical Center, Dallas, TX 75390-8852, United States

**Keywords:** prostate adenocarcinoma, colorectal adenocarcinoma, metastatic cancer, molecular diagnostics, precision oncology

## Abstract

Prostate and colorectal adenocarcinoma are among the most common primary cancer diagnoses in the United States, with 29% of new cancer diagnoses among adult men in 2024 expected to arise from the prostate and another 15% across men and women being colorectal in origin. Given therapeutic advancements leading to improved survival, individuals with prostate cancer have the highest estimated probability of concurrent secondary primary malignancy. This represents a clinical dilemma as the treatment for each is distinct and impacted by histology, stage, and molecular findings. Herein, we describe a patient with significant comorbidities, found to have simultaneous metastatic prostate and colorectal adenocarcinomas, who achieved sustained complete remission of liver and lung metastases with a chemohormonal regimen.

**Key Points**

- The case highlights the importance of utilizing advanced imaging techniques, morphologic analysis, pathology, and molecular profiling to overcome diagnostic challenges in managing complex cases of dual primary cancers.
- It underscores the challenges of relying solely on clinical guidelines and trial-based protocols, particularly when dealing with stage 4 double primary malignancies that are often underrepresented in most clinical trials, requiring a more flexible, individualized approach.
- When a combination therapy for two different cancers is not feasible, the case emphasizes the potential benefit of using a single regimen that can target both cancers, offering a “one stone, two birds” approach to treatment.

## Patient presentation

**Figure 1.**
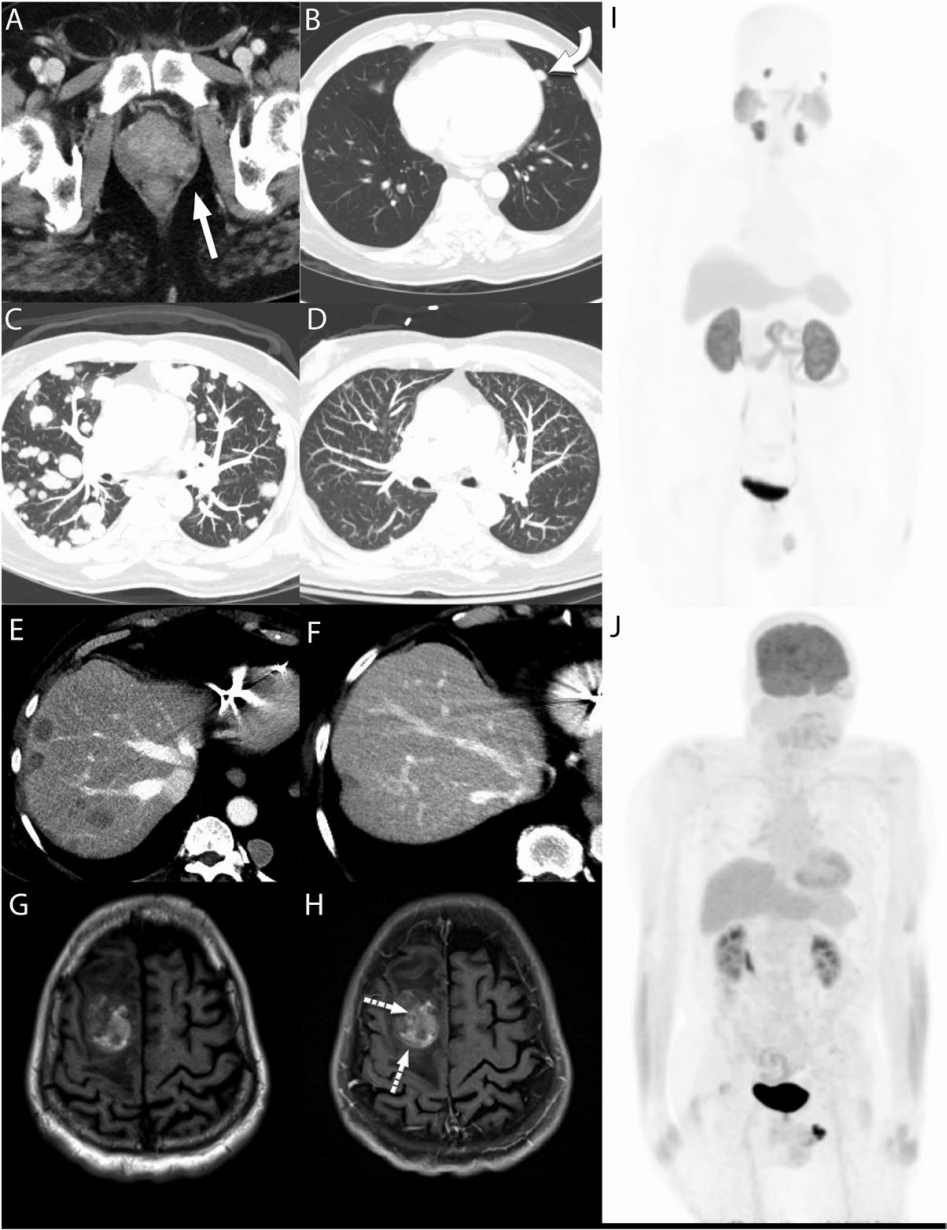
(A) Baseline axial CT image showing heterogenous enhancement of the prostate. (B) Axial CT image demonstrating a dominant 10 mm lingular nodule suspicious for metastatic disease. (C) Axial chest CT image 2 months after baseline demonstrating marked progression of pulmonary metastatic nodules. (D) Axial chest CT image 10 months after baseline with near resolution of pulmonary nodules with some residual opacities likely representing post treatment change. (E) Axial CT image demonstrating multiple progressive liver metastasis 2 months after baseline CT. (F) Axial CT image demonstrating resolution of liver metastasis 10 months after baseline. (G) Pre-contrast axial T1 image demonstrating a hemorrhagic mass in the right frontal lobe with associated mass effect. (H) Post-contrast axial T1 image demonstrating subtle enhancement of the mass that was suspicious for metastasis. (I) MIP image from Pylarify PET/CT with no appreciable abnormal uptake in the lungs or liver 7 months after diagnosis. (J) MIP image from FDG PET/CT 8 months after diagnosis demonstrating no appreciable suspicious uptake. Lung nodules were decreased in size and demonstrated low level non-specific uptake.

A 70-year-old male with non-ischemic cardiomyopathy and chronic systolic heart failure requiring cardiac resynchronization therapy defibrillator (CRT-D) placement in India presented to the emergency department with dysuria, urinary retention, rectal pain and marked deconditioning. Computed tomography (CT) imaging was remarkable for a heterogeneously enhancing prostate and multiple bibasilar pulmonary nodules measuring up to 10 mm. **Figure 1A,B**. There was concern for metastatic prostate cancer, but the prostate-specific antigen (PSA) level was only modestly elevated at 6.4 ng/mL. The patient was discharged and referred to Urology for follow up.

The patient returned to the emergency department approximately one month later, with worsening rectal pain and bleeding. CT of the chest demonstrated significant interval progression of innumerous well circumscribed bilateral solid cannonball pulmonary nodules of various sizes, now seen in all lobes of the lung, measuring up to 2.2 cm. A repeat CT abdomen also showed new hypoattenuating lesions in segment VI of the liver worrisome for further metastatic spread. **Figure 1C,E**

## Molecular Tumor Board

**Figure 2.**
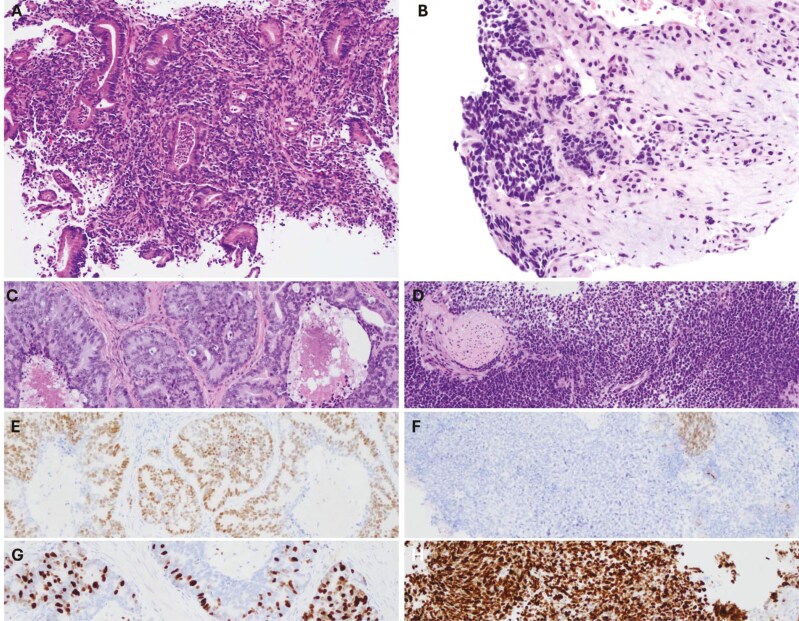
The biopsy of the mass in the ascending colon revealed a moderately differentiated invasive colorectal adenocarcinoma (A). Subsequent liver biopsy demonstrated a high-grade carcinoma (B), exhibiting different morphology from his colonic mass. Further investigation of his prostate by transurethral resection demonstrated prostate adenocarcinoma (C) and a small portion of poorly differentiated components (D), supported by NKX3.1 expression (D and F). Ki67 labelling index was low (5-30%) in conventional (G) and high (80%) in poorly differentiated portion (H).

A colonoscopy showed an infiltrating, non-obstructive mass 2.0 cm long within the ascending colon. Biopsy of this revealed moderately differentiated invasive colorectal adenocarcinoma **Figure 2A** with KRAS^G12D^ and SMAD4^C363Y^ mutations captured on next-generation sequencing. Additional biopsies were taken of the liver lesions, revealing a poorly differentiated carcinoma **Figure 2B**.

Given significant pain, obstructive urinary symptoms and possible abscess on imaging, the patient underwent transurethral resection of the prostate (TURP) with diagnostic and therapeutic intent, revealing prostate adenocarcinoma **Figure 2C** with the expression of NKX3.1 **Figure 2D** and 5-30% of Ki67 labelling index **Figure 2G** with small separate portions of poorly differentiation component **Figure 2D**, which lacked expression of NKX3.1 **Figure 2F** and demonstrated high proliferative index (80% of Ki67 labelling index) **Figure 2H**. INSM1, chromogranin and synaptophysin immunostains were negative in two components. Despite having no personal or family history of cancer, the patient was referred for germline testing which returned negative. Eventual molecular characterization of the prostate primary revealed intact mismatch repair with mutations of BRCA2, APC, RB1, CDKN1B and TMPRSS2-ERG **Table 1**.

**Table 1:** Molecular profile of individual tumor /metastasis biopsies

COLON	PROSTATE	BRAIN
Microsatellite indeterminate	Microsatellite Stable	Microsatellite stable
TMB indeterminate	TMB 8 mut/Mb	TMB 7 mut/Mb
KRAS G12D	BRCA2 S1172	BRCA2 S1172
NRAS wildtype	APC P2486fs*30	APC loss exons 2-5
SMAD4 C363Y	CDKN1B rearrangement intron 1	EP300 Q561
	RB1 loss exons 12-16	RB1 loss exons 12-16
	TMPRSS2-ERG fusion	TMPRSS2-ERG fusion
		HRD signature positive

Admission was further complicated by cerebrovascular accident of the left occipital lobe, although neuroimaging ruled out the presence of brain metastases. Following discussions between a multidisciplinary team and the patient, he began systemic therapy with 5-fluorouracil, leucovorin, and oxaliplatin combination chemotherapy (FOLFOX).

Shortly thereafter, pathology taken from the liver biopsy underwent re-review by multidisciplinary tumor board utilizing side-by-side comparisons of lesions in the colon, liver, and prostate. The poorly differentiated high-grade tumor in the liver was histologically identical to the poorly differentiated component in the prostate, with multiple shared markers of interest. This led to the conclusion that the liver lesion was a metastasis from the poorly differentiated elements of his prostate cancer. Considering this, the patient started on androgen deprivation therapy (ADT) with bicalutamide followed by leuprolide injections.

## Using molecular and radiologic data to guide treatment of concurrent metastatic cancer

In an aging population, the co-occurrence of malignancies is increasingly common and requires nuanced decision-making between multiple specialists at time of diagnosis and throughout treatment.^1-3^

Despite advances in histopathology, determination of tumor type in poorly differentiated specimens remains highly challenging. In our case, discussion with pathology indicated the prostate biopsy contained both conventional adenocarcinoma and a subgroup of poorly differentiated carcinoma. However, immunohistochemical markers for neuroendocrine differentiation were negative and there was limited tissue for further molecular analysis. At first review, the liver biopsy was reported as colon cancer, however later the diagnosis was revised based on morphologic similarity to the subgroup of poorly differentiated prostate cancer. In particular, both the prostate and liver biopsies revealed a high proliferation index of greater than 80%.

Our case further underscores the utility of NGS and molecular markers in challenging cancer diagnoses, especially when traditional histopathological methods are not definitive. The TMPRSS2-ERG fusion served as a key diagnostic tool in confirming the diagnosis of aggressive prostate cancer and suggesting a metastatic process to the liver, despite initial morphological confusion. The TMPRSS2-ERG fusion is a common translocation specific to prostate cancer, with some studies demonstrating an association with advanced stage disease upon diagnosis.^4^ Unfortunately, following histopathology there was inadequate liver tissue available for sequencing.

There was uncertainty regarding the primary cancer source of the multiple large metastatic lung lesions. Colon cancer lung metastases are typically characterized by multiple, well-circumscribed, cannonball-like nodules^5^ while those originating from prostate present with smaller, ill-defined nodules and concurrent intrathoracic lymph node involvement.^6^ Although a confirmatory lung biopsy would have delineated the true stage of the underlying colon adenocarcinoma, his limited cardiac reserve and recent CVA made him a poor candidate for such a procedure. Instead, both PSMA and FDG PET scans were obtained to leverage the specificity of PSMA in prostate cancer to help differentiate and localize the origin of metastatic disease.

With concurrent primaries, it is important to assess disease and symptomatic burden to decide about treatment strategy. Due to the extent of disease, a concurrent treatment strategy targeting both primary malignancies was preferred over sequential. Assessing patient frailty was paramount to the clinician’s decision to proceed with a regimen of FOLFOX and ADT, despite guideline recommendations for individual stage four disease to administer FOLFOX for metastatic colon cancer and concomitant carboplatin plus cabazitaxel for poorly differentiated metastatic prostate cancer, respectively. As guideline-based practice is largely governed by clinical trials and these often exclude patients with dual diagnoses, we may turn to experiential data, such as the case described by Tian et al., where a patient with synchronous HER2 + gastric and urothelial cancer initially received chemotherapy and trastuzumab, followed by nivolumab and trastuzumab due to adverse effects and recurrence of bladder cancer. This combination successfully controlled both cancers and improved the patient’s quality of life.^7^

Our patient initially presented with significantly compromised cardiorespiratory reserve and poor performance status. It is therefore highly unlikely he would have tolerated the recommended dual multiagent regimen without significant toxicity. Indeed, The American Society of Clinical Oncology (ASCO) guidelines emphasize the importance of a comprehensive geriatric assessment to identify vulnerabilities in older patients and accordingly, high-risk patients may benefit from reduced intensity treatments or single-agent therapies to mitigate these risks.^8^ In lieu of this, identifying a regimen with overlapping targets may be an ideal route.

Given the dual diagnosis and somatic p. S1172 BRCA2 mutation status of prostate cancer, a decision was made to administer FOLFOX and ADT. Our patient possessed a variant allele frequency (VAF) of 57.4% and HRD (Homologous Recombination Deficiency) score of 0.94, which increased clinical suspicion for chemosensitivity. In patients with prostate cancer possessing BRCA2 mutation, there is a growing body of evidence suggesting sensitivity to platinum-based chemotherapy.^9^ In a study by Cheng et al. several patients with metastatic castration-resistant prostate cancer (mCRPC) and biallelic inactivation of BRCA2 achieved an exceptional response to platinum-based chemotherapy.^10^ Similarly, Slootbeek et al. reported patients with mCRPC harboring BRCA2 mutations demonstrated positive biochemical responses to carboplatin, with significant declines in prostate-specific antigen (PSA) levels and objective radiographic responses.^11^ Together these findings suggest BRCA2 mutations in prostate cancer predict a favorable response to platinum-based chemotherapy, including oxaliplatin. Like 5-FU, oxaliplatin displays inhibitory activity of thymidylate synthase, preventing effective nucleic acid synthesis and cellular division. Although oxaliplatin-centric regimens have not been established as standard of care in prostate cancer, there have been multiple early-phase trials highlighting its utility with fluoropyrimidine agents in treating refractory prostate cancer with improved response and progression free survival, irrespective of BRCA status.^12,13^ Alternatively, in a subgroup of the ProBio trial, mCRPC patients with DNA repair deficiency (8 of 42 patients with BRCA1/2 alterations) had inferior progression free survival with carboplatin compared to standard of care therapy (survival time ratio of 0.68, 90% CI 0.45-1.07), suggesting the relationship between HRD and platinum response is more nuanced than previously suggested.^14^

## Patient update

The patient was originally referred for a biopsy of the lung lesions to determine their primary origin, but it was not performed due to his limited cardiac reserve and recent CVA. Instead, PSMA PET and fluorodeoxyglucose-positron emission tomography (FDG PET) scan returned without focal uptake at the liver, colon, or lung, indicative of a complete metastatic response **Figure 1**I,J. Mild PSMA and FDG uptake remained in the prostate. Of note, the PSMA PET/CT was obtained after 4 cycles of FOLFOX and after one month of chemical castration. The patient eventually completed 12 cycles of FOLFOX with imaging confirming sustained response **Figure 1** D,F.

Eight months after initiation of chemotherapy, the patient presented with right-sided weakness. Brain MRI revealed an intraparenchymal hematoma with mass, prompting a partial craniotomy with hematoma evacuation **Figure 1**G,H. Pathology taken during the procedure returned with metastatic high-grade carcinoma **Table 2**. He was evaluated by radiation oncology for post –operative SBRT to the surgery bed. Remarkably, following intervention the patient experienced resolution of neurologic deficits. Prior to this presentation, the patient had been on abiraterone and prednisone for three weeks. This was held during admission and upon discharge, the patient opted against its continuation, in favor of the PARP inhibitor olaparib. Twelve months after initial diagnosis, restaging scans show no evidence of disease recurrence in his lungs and liver, PSA remains undetectable, and a repeat colonoscopy showed no abnormality. Importantly, the patient’s quality of life improved drastically, having originally presented wheelchair-bound and suffering from debilitating symptoms for over a year before diagnosis, now free of urinary symptoms, ambulating without assistance and visiting the gym multiple times a week.

**Table 2.** The immunohistochemical (IHC) findings from tumor biopsies

	Colon	Prostate	Liver	Brain
KI67	n/a	90%	90%	80%
CK7	Negative	negative	n/a	negative
CK20	Positive	negative	negative	negative
CDX2	Positive	n/a	positive	positive
TTF-1	n/a	n/a	negative	negative
NKX3.1	negative	positive	negative	negative
GATA3	n/a	negative	negative	negative
CAM5.2	n/a	n/a	positive	positive
INSM-1	n/a	negative	negative	n/a
CHROMOGRANIN	n/a	negative	n/a	negative
SYNAPTOPHYSIN	n/a	negative	negative	negative
PSA	n/a	positive	n/a	negative
P53	n/a	n/a	n/a	positive
P63	n/a	positive	n/a	positive
MMR	Intact	n/a	n/a	n/a

## Conclusions

Managing an elderly patient with concurrent metastatic prostate and colon cancer required an adaptive, multidisciplinary strategy. This case highlights diagnostic challenges posed by indeterminate lung lesions and complex liver pathology in a patient with two primary cancers. The therapeutic approach, guided by molecular insights and the patient’s tolerance, underscores the necessity of flexible, tumor-agnostic strategies that prioritize patient well-being when traditional guidelines are impractical. By consolidating treatment into a single regimen, we balanced effective cancer control with a lower risk of toxicity, addressing both the biological needs of the tumors and the clinical needs of the patient.

In the future, access to non-invasive diagnostic tools and broader clinical trial inclusion criteria for patients with multiple malignancies and extensive comorbidities will be essential. As oncology continues to advance, embracing adaptable, multidisciplinary care will remain crucial in addressing the intricate needs of complex, elderly patients who defy conventional diagnostic and therapeutic norms.

## Data Availability

Data supporting this article is provided in the text and figures. Additional information is available from the authors upon reasonable request.
